# Love, Work, and Striving for the Self in Balance: Anaclitic and Introjective Patients’ Experiences of Change in Psychoanalysis

**DOI:** 10.3389/fpsyg.2020.00144

**Published:** 2020-02-14

**Authors:** Andrzej Werbart, Annelie Bergstedt, Sonja Levander

**Affiliations:** Department of Psychology, Faculty of Social Sciences, Stockholm University, Stockholm, Sweden

**Keywords:** psychoanalysis, patient perspective, dimensions of change, personality configurations, thematic analysis

## Abstract

One of the most famous quotations credited to Freud is that, when asked what he thought a psychologically healthy person should be able to do, he said: “to love and to work.” A central goal in psychoanalytic treatment is to bring about changes in basic, mostly unconscious, mental structures. The aim of this study was to investigate, applying an inductive thematic analysis, the experiences anaclitic and introjective patients have had of change after psychoanalysis with regard to the domains *Love and Relationships* and *Work and Achievements*. Analyzing patient interviews, we identified a third domain of experienced changes, *The Self*, which refers to increased self-understanding, self-acceptance, and self-care rather than an improved dynamic balance between love and work. All patients experienced several positive changes in their lives during and after psychoanalysis. We also found distinctive patterns that appear to be closely linked to the patients’ initial personality orientation with regard to relationships and achievements. Generally, the patients described symmetrical, but opposite, change processes within the two specific domains of Love and Work. For the anaclitic patients, this indicated a movement inward in the domain of Love (from an excessive preoccupation with issues of their relationship with others toward more distinct self-boundaries and increased agency) and outward in the domain of Work (from unenterprising toward becoming more outgoing and daring). For the introjective patients, this pointed to a reverse movement outward in the domain of Love (from an excessive preoccupation with issues of autonomy toward increased responsiveness to others and desire to be establish close, mutual relationships) and inward in the domain of Work (from an excessive orientation on achievements toward increased becoming more grounded in their own feelings, needs, and desires). In conclusion, patients in both groups have experienced a reduced preoccupation with issues related to their initially predominant personality dimension (relatedness or self-definition) and increased receptivity to needs typical for the complementary dimension. These changes seem to be mediated by changes in the domain of *The Self*. Our study suggests the clinical relevance of focusing the therapeutic work on fostering a better and more dynamic balance between love and work, relatedness, or self-definition.

## Introduction

When Freud was once asked what he thought a psychologically healthy person should be able to do well, he was reported to answer: “lieben und arbeiten” (to love and to work). The phrase was quoted in 1950 by Erik Homburger Erikson (1963, p. 265) but cannot be found in Freud’s writings (Elms, 2001). However, the maxim was not foreign to Freud (1961). For example, in *Civilization and its discontents* (1930/1961, p. 101), he stated: “The communal life of human being had… a twofold foundation: the compulsion to work… and the power of love…” The idea of a balance between love and work is also central to several influential theories of psychological maturity and well-being (e.g. Maslow, 1954; Rogers, 1961; Erikson, 1963). As suggested in 1980 by the grand old man of psychotherapy research, Hans Strupp (1980) the basic commitment of psychoanalysis is “to the dual goal of personal freedom and human relatedness” (p. 399). In a seminal paper, Hazan and Shaver (1990) linked secure attachment with the ability to find a balance between love and work.

According to Blatt’s “double helix” model, psychological development is a lifelong synergic interaction between two fundamental dimensions in human experiences: relatedness and self-definition (Blatt, 2008; Blatt and Luyten, 2009; Luyten and Blatt, 2011). Following this model, psychological well-being involves both meaningful relationships with significant others as well as autonomy and differentiation between the self and others—in other words, a mature balance between affiliative and achievement-related needs. Most individuals, within a normal developmental variance, tend to emphasize one or the other dimension and differ in their relative emphases on issues concerning love and work. Different forms of psychopathology reflect an excessive and disturbed preoccupation with one dimension at the expense of the other. The anaclitic configuration is connected with difficulties in close relationships and attachment anxiety, while the introjective configuration is connected with excessive demands for achievement and perfectionism as well as attachment avoidance (Luyten and Blatt, 2013). Accordingly, anaclitic depression centers on feelings of loneliness, abandonment, and neglect, and introjective depression focuses on issues of self-worth and feelings of failure and guilt (Blatt, 1974, 2004; Blatt and Luyten, 2009).

Consistent results from several psychotherapy studies have indicated that anaclitic and introjective patients are differentially responsive to different aspects of the psychotherapy process and express therapeutic progress in different ways (Blatt, 1992, 2007, 2011; Blatt and Ford, 1994; Blatt and Shahar, 2004; Blatt et al., 2010; Luyten and Blatt, 2013; Lowyck et al., 2016). Anaclitic patients respond to the supportive dimensions of the treatment process, whereas introjective patients have a better therapeutic response to the exploratory-interpretative dimensions of the treatment process. Anaclitic patients change primarily in the quality of their interpersonal relations, whereas therapeutic change in introjective patients occurs primarily in the area of their manifest symptoms and in the level of cognitive functioning. Thus, both groups of patients display therapeutic change along dimensions congruent with their predominant personality organization.

In a quantitative multi-case study, Werbart and Forsström (2014) showed that both anaclitic and introjective patients in psychoanalysis maintained their characteristic personality style. However, all initially anaclitic patients and less than half of the initially introjective patients met the criterion of improved anaclitic–introjective balance post-treatment and/or follow-up. Noticeably, symptom reduction was accompanied by more mature integration of anaclitic and introjective personality dimensions in the anaclitic cases, whereas the introjective patients could show symptom reduction without improved anaclitic–introjective balance. Similarly, in a quantitative replication study of young adults in psychoanalytic psychotherapy (Werbart et al., 2017), the anaclitic patients showed better balance between relatedness and self-definition post-treatment, whereas this improvement was not significant in the introjective group. Still, on a descriptive level, both studies showed that the anaclitic as well as the introjective patients showed a mean score reduction on the predominant personality dimension and an increased mean score on the opposite dimension from pretreatment to termination. This indicates that psychoanalytically oriented treatments contribute to better anaclitic–introjective balance: decreased preoccupation with issues related to the predominant personality dimension and increased responsiveness to the issues characteristic for the complementary dimension.

Furthermore, qualitative studies demonstrated that the anaclitic and the introjective patients in psychoanalysis differ in their experiences of helpful and hindering factors in psychoanalysis and in their experiences of the analyst (Levander and Werbart, 2012; Werbart and Levander, 2016). The anaclitic patients talked of trust and a wish for support from their analyst, whereas the introjective patients wanted help with their control and in maintaining distance from others. The anaclitic patients could experience the firm time frames as a hindrance, whereas the introjective patients saw their own shortcomings but also the analysts’ ways of working as the main hindrances (Levander and Werbart, 2012). In another sample, patients’ pictures of their analysts after termination bore the stamp of the patients’ predominant personality orientation. Anaclitic patients described both positive and negative aspects of their relationship, whereas introjective patients focused on both positive and negative aspects of the therapist as a separate individual (Werbart and Levander, 2016).

To sum up, previous studies open up for further questions. Is the therapeutic change in the anaclitic and introjective patients mainly congruent with their predominant personality orientation? What about the changes in the opposite dimension? Do the patients themselves experience better balance between relatedness and self-definition? Prompted by these issues, in the present study, we have focused on the anaclitic and the introjective patients’ experienced changes in the areas of love and work. “Love and Relationships” refers here to the patients’ coping with their affiliative needs and interpersonal bonds; and “Work and Achievements” refers to their coping with strivings for autonomy, performances, and self-demands. How do the anaclitic and the introjective patients describe and value their changes after psychoanalysis in these two domains? What is distinguishing and what is common in their subjective experiences of change?

## Materials and Methods

### Data Collection

The psychoanalyses included in the present study were conducted within the ordinary work of the former Institute of Psychotherapy, Stockholm County Council, Sweden, where subsidized psychotherapy was provided for people with various psychological problems. Consecutive cases of psychoanalyses were included during a 5-year period between 1997 and 2002. The 18 patients were referred to psychoanalysis from psychiatric outpatient clinics. The data were collected between 1997 and 2008 at intake, termination, and 2-year follow-up and included the following instruments: the Symptom Checklist-90-R (SCL-90; Derogatis, 1994), the Structural Analysis of Social Behavior (SASB) Intrex Questionnaire (Benjamin, 1987), and the Sense of Coherence (SOC) Scale (Antonovsky, 1987). The data were previously used in two studies of changes in anaclitic–introjective personality dimensions following psychoanalysis (Werbart and Forsström, 2014; Werbart and Levander, 2016). The present study is based on 14 patient interviews at termination and 12 patient interviews at 2-year follow-up (26 patient interviews in total). At follow-up, one former patient was unreachable and another one was deceased after prolonged illness (one of the cases was assessed as initially anaclitic and the other as introjective). Additionally, 14 analyst interviews at termination were included in the assessments of the patients’ initial personality configurations.

### Participants

The attrition was 22%: three patients declined participation in the data collection and one patient started low-frequent psychotherapy instead of psychoanalysis (defined as a treatment conducted by a psychoanalyst with a frequency of four to five times a week). Thus, the sample comprised 14 cases of psychoanalysis. The mean age of the patients at inclusion was 33 years (range 25–46; *SD* 7.1), and 12 of them were women. All patients but one had received previous psychiatric treatment; 12 patients had also prior experience of psychotherapy. The patients’ psychiatric diagnoses were not accessible to us. However, their most common presenting problems were depression and anxiety; anorexia, psychosomatic symptoms, self-harm, and protracted crisis reactions were also represented. Four patients were on psychopharmacological medication at the start of psychoanalysis; two of them continued medication at both termination and 2-year follow-up, while a further two resumed medication at follow-up.

The 14 included analyses lasted for between 46 and 85 months (*M* = 61; *SD* 14.7) with a frequency of four sessions a week and were conducted by eight highly experienced female analysts. On the group level, the patients improved on all measures, with effect sizes comparable to mean effect sizes reported in a meta-analysis of studies regarding the effectiveness of psychoanalytic treatments (de Maat et al., 2013), and the improvements continued after termination. Most patients showed positive outcomes in terms of symptom reduction (SCL-90) and improved quality of life (SOC), and the proportion of patients showing clinically significant change was comparable to data presented by de Maat et al. (2013), while changes in positive and negative self-concept (SASB) were more limited. For more detailed outcome data, see Werbart and Forsström (2014) and Werbart and Levander (2016).

### Change After Psychotherapy Interviews

The applied CHAP-protocol (Change after Psychotherapy; Sandell, 2005, 2015; Sandell and Wilczek, 2016) is especially designed to capture the complexity patients’ subjective experiences of change during and after psychotherapy. The patients are asked about in what ways they have changed during or after the therapy, how they feel now and how their present life situation is in comparison with their pre-treatment life, what has improved, what is unchanged or worse, and what contributed to these outcomes. The analysts were asked corresponding questions about their patients.

### Assessment of Personality Configuration

Assessment of the patients’ initial personality configurations followed the procedure of *Prototype Matching of Anaclitic-Introjective Personality Configuration* (PMAI; Werbart and Forsström, 2014; Werbart and Levander, 2016). On the basis of CHAP interviews, two raters assessed the extent to which the presentations of the patient’s problems and life circumstances pre-treatment matched prototype descriptions of the anaclitic and introjective personality orientation. Due to the research design, the initial personality configuration was assessed based on the CHAP descriptions of the pre-treatment situation. To enhance the validity of these ratings, both patient and analyst CHAP interviews at termination were used in each case. The raters, two students at an advanced psychotherapy training program, were trained in the PMAI assessment. The inter-rater reliability was satisfactory (ICC = 0.65). Cases were assessed as initially anaclitic or introjective based on consensus ratings and following the highest pre-treatment score on one of the two dimensions. This resulted in seven patients classified as initially predominately anaclitic and the remaining seven patients as predominately introjective (Werbart and Forsström, 2014; Werbart and Levander, 2016).

### Comparative Thematic Analysis

**FIGURE 1:**
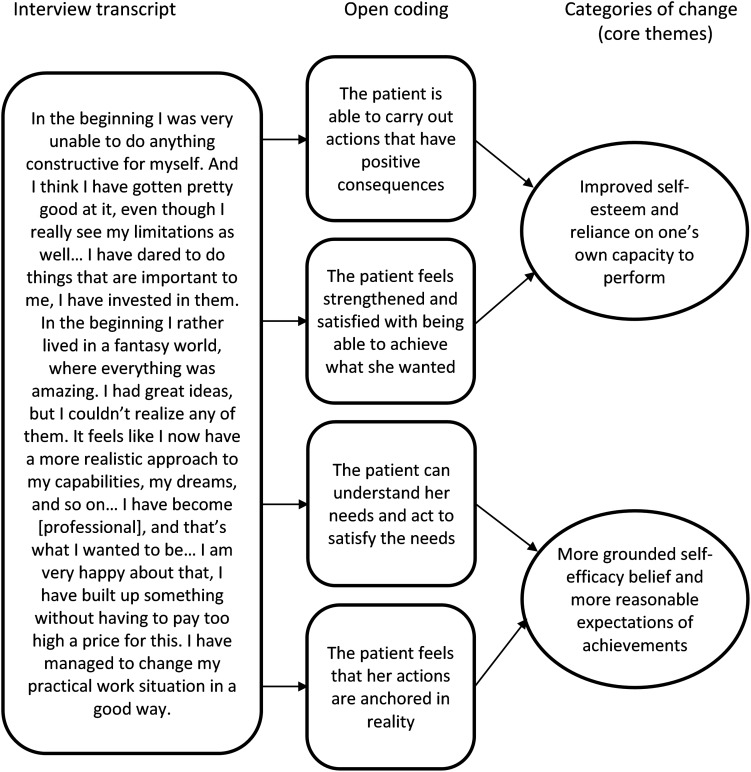
Data analysis: steps from interview transcripts to categories of change (core themes).

A first step in the data analysis was to select all utterances about experienced changes from the CHAP interviews. The patients’ descriptions of changes were categorized using an inductive thematic analysis (Boyatzis, 1998; Braun and Clarke, 2006), a method also called “inductive clustering” (Miles and Huberman, 1994). At an early stage of data analysis, it turned out that the described changes displayed only negligible differences between the interviews at termination and at the 2-year follow-up. This means that the same themes emerged at both occasions, even if the degree of elaboration could vary. Accordingly, the categorization was performed, combining both interviews for each case (in the two cases of attrition at follow-up only the interviews at termination could be used). Following the aims of the present study, the patients’ experiences of changes were analyzed separately in the thematic domains *Love and Relationships* and *Work and Achievement*. A new thematic domain emerged in the analysis process: *The Self*, comprising themes of experienced change outside the dichotomy of Love and Work. An illustration of the data-driven coding process is provided in Figure 1.

The categorization of experienced changes was performed blindly for the patients’ personality configuration. The total interview material was analyzed jointly by two judges (the second author and another student of psychology), following the rules for consensual qualitative research (Hill et al., 2005), and audited by the first author. The step-by-step procedure of thematic analysis (Braun and Clarke, 2006) started with close reading of the 26 interview transcripts, searching for meanings and patterns in the descriptions of experienced changes. In the next step, relevant segments of transcripts were identified and the two judges generated initial codes. Subsequently, the similar or closely related codes were assembled into core themes (categories of experienced changes) and labeled. The themes were repeatedly revised, compared to the data, and refined in consensus discussions. In the process of elaborating descriptions of each category and sorting the categories into the two predetermined domains, the new thematic domain of *The Self* was formed.

After the list of core themes was established, the data were decoded, and the anaclitic and the introjective patients’ experiences of changes were compared. Results within each domain were organized in descending frequency of cases in each domain. Frequencies are reported separately for the anaclitic and introjective group as well as for the total sample, following nomenclature from Hill et al. (2005). The label “general” applies to all, or all but one case, “typical” to more than half of the patients, and “variant” to at least two and up to half of patients. Due to the small numbers, a difference of two or more cases together with different labels was considered as a between-group difference (cf., Werbart and Levander, 2016).

## Results

Thematic analysis of the patients’ narratives of change resulted in 12 categories (core themes) within the domains *Love and Relationships* and *Work and Achievements*. However, in analyzing patient interviews, four additional themes emerged, and together they constitute a third domain, *The Self*, which refers to changes that are directed toward the individual’s experience of being a particular person and a subject of own reflexive consciousness rather than changes in the dynamic balance between love and work. The categories of experienced change are reported for the initially anaclitic and introjective patients and for the total sample in Table 1 and presented below. The change categories are illustrated below by verbatim quotations from interviews with patients from both groups. All patients have been given assumed names with the initial letters “A” for the anaclitic and “I” for the introjective patients. Quotations from interviews both at termination and follow-up are included. The presentation of each domain concludes with a between-group comparison.

**TABLE 1:** Categories of experienced changes among anaclitic and introjective patients in psychoanalysis.

**Category**	**Anaclitic**		**Introjective**		**Total**	
	**(*n* = 7)**	**Label**	**(*n* = 7)**	**Label**	**(*n* = 14)**	**Label**
**Domain 1: Love and Relationships**						
1.1. Increased Relational Insight Fostering New Ways of Relating	**3**	**Variant**	**5**	**Typical**	8	Typical
1.2. Increased Trust in Others, Relational Self-Confidence, and Openness	**4**	**Typical**	**2**	**Variant**	6	Variant
1.3. Improved Ability to Deal With Difficulties and Conflicts in Close Relationships	**4**	**Typical**	**2**	**Variant**	6	Variant
1.4. Increased Ability to Set Limits and Take Responsibility for Oneself	3	Variant	3	Variant	6	Variant
1.5. Increased Capacity for Intimacy and More Authentic and Meaningful Relationships	2	Variant	3	Variant	5	Variant
1.6. More Stable Self-Boundaries and Increased Independence in Relationships	**5**	**Typical**	**0**	**Absent**	5	Variant
1.7. Improved Ability to Express Own Feelings, Needs, and Wishes in Relation to Others	2	Variant	2	Variant	4	Variant
**Domain 2: Work and Achievements**						
2.1. Improved Self-Esteem and Reliance on One’s Own Capacity to Perform	**5**	**Typical**	**3**	**Variant**	8	Typical
2.2. More Grounded Self-Efficacy Belief and More Reasonable Expectations of Achievements	**2**	**Variant**	**5**	**Typical**	7	Variant
2.3. Increased Insight Into Achievement Related Patterns of Behavior	3	Variant	2	Variant	5	Variant
2.4. More Flexible Coping and Adjustment to Performance-Related Stress	**1**	**Rare**	**4**	**Typical**	5	Variant
2.5. Better Contact With Own Achievement-Related Needs and Increased Self-Determination	**0**	**Absent**	**3**	**Variant**	3	Variant
**Domain 3: The Self**						
3.1. More Mature and Nuanced Self-Understanding and Realistic View of Life	6	General	5	Typical	11	Typical
3.2. Enhanced Self-Regard and Ability to Care for Oneself	4	Typical	4	Typical	8	Typical
3.3. Increased Ability to Accept and Value Oneself	4	Typical	3	Variant	7	Variant
3.4. Improved Reflective Capacity, Experiential Processing and Resilience	4	Typical	3	Variant	7	Variant

**Note*. Frequencies of cases in each category (labeled following Hill et al., 2005) separately for the anaclitic and introjective group (General: six to seven cases; Typical: four to five cases; Variant: two to three cases; Rare: one case) and totally (General: 13–14 cases; Typical: 8–12 cases; Variant: two to seven cases; Rare: one case). Between-group differences of two or more cases and different labels are in bold (cf., Werbart and Levander, 2016).*

### 1. Love and Relationships

#### 1.1. Increased Relational Insight Fostering New Ways of Relating

As a variant, anaclitic patients and, typically, introjective patients described how an increased reflective self-examination of own relational patterns across different social contexts guided them to more adaptive ways of relating to others. New interpersonal awareness and self-observing ability helped them to recognize problematic patterns and to relate to others in more mindful and constructive ways. For example, they could gain a more profound understanding of how own fears of rejection and negative assumptions previously had influenced them to “act blindly” or “do the opposite.” Women could feel being attracted to different kinds of men than before, the anaclitic patients no longer seeking unconditional love and the introjective patients no longer being tempted by men who hurt them. Patients in both groups experienced an improved capacity to deepen existing relationships and to build new ones.

#### 1.2. Increased Trust in Others, Relational Self-Confidence, and Openness

Typically, anaclitic patients and, as a variant, introjective patients described how processing previous traumas and suffering offered an opportunity to build up an inner security in interactions with others. They told about feeling less alienated, challenging old beliefs about themselves in relationships, and daring to long for contact with others. They described developing a more solid self-confidence and being less governed by fear and shame, which enabled them to be more open and receptive toward others and to experience the joy of having others in their lives. They experienced growing reliance on others’ ability to meet their needs and on their own ability to seek help and share difficulties.

#### 1.3. Improved Ability to Deal With Difficulties and Conflicts in Close Relationships

Typically, anaclitic patients and, as a variant, introjective patients described how psychoanalysis helped them to recognize different viewpoints in situations, adjust high expectations of significant others, and question their ways of interacting with others. Exploring patterns of interpersonal difficulties gave them a greater relational self-awareness and an increased tolerance for conflicts. They learned more constructive ways of dealing with disagreements and negative emotion. Furthermore, they developed a greater ability to become reconciled with their past relational patterns, accept circumstances they could not change, and to have more fun with people around them.

#### 1.4. Increased Ability to Set Limits and Take Responsibility for Oneself

As a variant, patients in both groups described taking a more active part in how their relationships develop. This included being better at seeing their own role, having less feelings of victimhood, and assuming more responsibility in relationships. Patients also came to act in more self-regarding ways, recognizing their own needs and boundaries as legitimate sources of action. Patients described changes such as becoming less reactive and more in control of things. They gained courage to stand up for themselves, to recognize their own determination and power to affect their relationships, and, in that way, to take better care of themselves in relationships.

#### 1.5. Increased Capacity for Intimacy and More Authentic and Meaningful Relationships

As a variant, patients in both groups described an increased openness for own affiliative needs, daring to feel and express love and vulnerability as well as to disclose themselves and confide in others. They felt they developed an ability to handle closeness and to deepen their relationships based on intimacy and reciprocity. Furthermore, they became more susceptible to the needs of others and more accessible to their friends, parents, or partners.

#### 1.6. More Stable Self-Boundaries and Increased Independence in Relationships

Anaclitic patients typically described how they developed more pronounced and stable self-boundaries. What led them to psychoanalysis was lacking a sense of inner kernel, being excessively preoccupied with mirroring themselves in others, seeking approval, and having an infantile need of being taken care of by their partners. Becoming more in touch with their emotions and increasing their self-knowledge enabled them to be more grounded in themselves. Patients described how gaining a better sense of own identity and independence fostered new ways of being with others. This theme was absent in the narratives of introjective patients.

#### 1.7. Improved Ability to Express Own Feelings, Needs, and Wishes in Relation to Others

As a variant, patients in both groups described a new awareness of previously suppressed emotions. They felt that it was more okay to be afraid or angry and learned to recognize what they felt and needed in relation to others, to react, let go of emotions, and to speak up instead of “becoming paralyzed” or to “stash away feelings.” This seems to have made them more able to show who they were without subsequent feelings of guilt or anguish and to dare to be honest with themselves and with others.

#### Comparison

Although both anaclitic and introjective patients described several similar positive changes within the domain *Love and Relationships*, we also found distinguishing patterns, closely linked to their initial personality orientation and the problems that brought them to analysis. Both anaclitic and introjective patients expressed an increased insight in own relational patterns and a new ability to challenge their perceptions of themselves and others. They felt they developed more adaptive ways of dealing with social situations, regulating closeness and distance, and assuming greater responsibility for themselves. Central to these processes was an increased trust and being more in contact with and accepting of their own feelings and affiliative needs. Nevertheless, we found four between-group differences in frequencies of categories within this domain (Table 1). More anaclitic than introjective patients reported increased trust in others and relational self-confidence, improved ability to deal with relational difficulties and conflicts, and more stable self-boundaries and increased independence. More introjective than anaclitic patients reported increased relational insight and new ways of relating.

Most importantly, not only the predominantly anaclitic patients but also the predominantly introjective patients described essential improvements in the domain *Love and Relationships.* However, there are some qualitative distinctions between the two groups as to the content and direction of these changes. The anaclitic patients described that, prior to analysis, they felt a lack of a stable identity and an inability to sort out and regulate their emotional reactions. They gave several examples of a development from an excessive preoccupation with how other people perceived them and what others had exposed them to and a great need of being approved, liked, and cared for by others, toward a greater recognition of their own behavior and the roles they tended to take in relationships. This indicated an overall movement *inward*, from an excessive neediness and preoccupation with others toward an increased interpersonal self-awareness, more distinct self-boundaries, increased agency, and emotional balance.

When I started in psychoanalysis, everything tended to be about my family and what other people had done to me. Some sort of huge disappointment. From that, it became much more about myself, going into myself and back to myself, what I was doing to myself, it was a very important progress. […] That was very difficult to discover, and it took a long time to deal with. [Amanda, termination]

[…] I am not so keen to “win” other people’s favor any longer. […] I think I dare to put my foot down more often now. Even if it’s not a major revolution, it makes me proud. […] Things seem to get less charged. When I don’t need to be in control in the same way, then it becomes less awkward to others if I speak up. If I want to draw a limit, then I do. That doesn’t mean I reject them for the rest of my life. Only “not here, and not now, I say no.” So I have changed. [Andrew, follow-up]

In contrast, the introjective patients’ descriptions of change focused on themes of control and independence. They described their avoidant or dismissive relational patterns prior to psychoanalysis due to their fears of rejection, feelings of inferiority and shame, and a profound inner sense of not deserving love and care. Inability to cope with intimacy and closeness resulted in loneliness, isolation, or relationships marked by emotional unavailability and lack of reciprocity. In psychoanalysis, they felt they gained an increased awareness of own avoidant patterns, became less governed by fears of being dependent or losing control, and were better able to handle intimacy and interdependency. They became capable of asking others for help and support, to care for their close relationships, and to be more present as parents, partners, and friends. Taken together, this indicated a movement *outward* from an excessive preoccupation with self-boundaries and autonomy toward greater acceptance of own affiliative needs, increased ability and desire to establish and deepen close, mutual relationships, and increasing responsiveness to others.

I started psychoanalysis and came to question my pattern with men. […] I had dated married men; I had lived with men who hurt me. And now, I have met a man who is incredibly caring and loving, he is completely different. This would never have attracted me before; it would have been boring and not so appealing. Anyway, it is an incredible change; I am fully convinced that I would never have met him if it wasn’t for psychoanalysis. It’s simply an incredible difference, this closeness, and to bear him actually seeing how sick I am, and to feel that it is okay, it was unthinkable before. [Irja, termination]

I didn’t want to say a word about myself to another person! […] The most difficult thing was that when something happened, when I ever needed to talk to someone, I couldn’t do it. When I tried, it made me feel even worse. I felt really bad about what I had told and how it could be perceived by others. Now, I can do this very well, I am able to open up and to talk about it, it’s virtually no problem at all. […] And the most amazing thing, I also thought, what a lot of amazing people out there, they didn’t exist before. And it is hardly because the people have changed, but only that I see them. [Inez, follow-up]

### 2. Work and Achievements

#### 2.1. Improved Self-Esteem and Reliance on One’s Own Capacity to Perform

This theme, typical for anaclitic and a variant for introjective patients, embodies descriptions of how psychoanalysis contributed to greater professional confidence. Patients described how they came to nuance their self-perception, challenging old convictions of their professional self and what they could attain in life. The felt they started daring to believe in themselves, discovered new options, and succeeded to make concrete changes in their lives, such as returning to work, finishing their studies, changing occupation, or setting up their own business. New perspectives on themselves and recognition of both capabilities and limitations made them feel liberated, more positive, and confident in possibilities to form their own future.

#### 2.2. More Grounded Self-Efficacy Belief and More Reasonable Expectations of Achievements

As a variant, anaclitic patients and typically, introjective patients described how previous excessive self-demands, need of control, and pursuit of performance turned into obstacles to personal development and growth and impaired their quality of life. In psychoanalysis, unrealistic expectations had been shut down and many illusions been abandoned. Patients described how they reappraised their ambitions and direction in life, becoming at peace with themselves. Being less hard on themselves, and able to set more realistic goals, patients could both manage to accomplish ambitions and find more pleasure in life.

#### 2.3. Increased Insight Into Achievement-Related Patterns of Behavior

As a variant, patients in both groups described gaining insight in own performance-related patterns of behavior, as well as an increased capacity to reflect on and to place their aspirations and difficulties in a context of their personal history. This helped them to establish a basis for professional initiative and determination, to prioritize differently, to widen their perceived scope of opportunities and to feel more as participants in their own lives.

#### 2.4. More Flexible Coping and Adjustment to Performance-Related Stress

The introjective patients typically described how they learned to moderate their need for control and how they became kinder and more generous to themselves, less governed by rigid self-demands and feelings of shame. They felt that they could cope better with strains and difficulties and develop more adaptive strategies to meet performance-related challenges. They learned to ask for help and to use people around them for support. The only anaclitic patient in this theme described that she became less sensitive, no longer avoided challenges, and was better at accepting shortcomings.

#### 2.5. Better Contact With Own Achievement-Related Needs and Increased Self-Determination

Only introjective patients described, as a variant, better contact with own work-related aspirations and needs, feeling less of a need to be at the center of attention, and becoming less directed by experienced expectations of others. They felt better anchored in themselves, with a greater sense of autonomy and self-assertiveness.

#### Comparison

Many of the changes, described in a similar way by both anaclitic and introjective patients, implied concrete and significant shifts in their lives. They could return to work, change occupation, apply for studies, or accomplish their education. Patients described improved professional confidence, having more realistic and nuanced expectations of achievements, recognizing capabilities as well as limitations, and obtaining tools to manage hardships and challenges in new, more constructive ways. In this domain, we also found four between-group differences (Table 1). More anaclitic patients described improved self-esteem and reliance on one’s own capacity to perform, whereas more introjective than anaclitic patients described a more grounded self-efficacy belief and expectations of achievements, better coping with performance-related stress, and better contact with own achievement-related needs.

Accordingly, not only the predominantly introjective, but also the predominantly anaclitic patients experienced crucial changes in the domain of *Work and Achievements.* However, the nature of their initial difficulties and the direction of these changes differed. Among anaclitic patients, fantasies and unrealistic expectations of achievements appeared to have contributed to psychological barriers leading to apathy, dejection, and lack of action. In psychoanalysis, they were able to strengthen their self-confidence and change perspective on their professional selves and what they could attain in life. Adjusting their goals and ambitions to be more realistic helped them to break passivity and not be as hindered by anxiety and fear of failure as before. The anaclitic patients described becoming more outgoing, courageous, and daring in their enterprises. Accordingly, their accounts of changes within the domain of *Work and Achievements* implied an overall expansive turn *outward* from states of passivity and hiding themselves toward daring to believe in themselves, be ready to face the world, and to take action.

I think I had an illusory picture of myself and what I had to achieve. That was why I didn’t manage to go out and perform. It always felt like a failure, because I had a preposterous picture of who I was. […] My expectations of every job were so high, so in the end I didn’t look for a job anymore. […] But at the end of psychoanalysis, I began to increasingly see myself in a more realistic way. I think I did it quite concretely. […] [My analyst] helped me achieve smaller goals, to cope with things. [Astrid, termination]

I have dared to do things that are important to me, I have invested in them. In the beginning, I rather lived in a fantasy world where everything was amazing. I had great ideas, but I couldn’t realize any of them. It feels like I now have a more realistic approach to my capabilities, my dreams, and so on. […] I have become [professional], and that’s what I wanted to be […] I am very happy about that, I have built up something without having to pay too high a price for this. I have managed to change my practical work situation in a good way. [Amanda, follow-up]

Introjective patients described that what brought them to psychoanalysis were excessive self-demands, perfectionism, and need of control. They felt that their rigidity, self-criticism, and striving for achievement and recognition resulted in high workloads, restless activity, performance-related anxiety, high internal stress, and an urge to constantly challenge themselves. In psychoanalysis, they came to realize how unreasonable expectations and their constant need of pushing themselves affected their quality of life. They described their changes in terms of wishing for a better life, learning to moderate their demands, reevaluating ambitions, gaining more flexible control over their lives, achieving greater calm, as well as living more slowly, thoughtfully, and in greater contact with their own needs and aspirations. Taken together, this resulted in increased stability and self-determination. The changes described by the introjective patients within this domain can be summarized as a turn *inward*, from an excessive orientation on achievements and prestige toward increased introspection and reflection, becoming more grounded in their own feelings, needs, and desires.

Yes, the turning point is my job. When I got the job I have today, it was extremely important. At the same time, I think psychoanalysis enabled me to take this job. That I could trust myself to the extent that I dared to act. […] [A]fter all, it is a very good incentive for yourself when you can handle things in life. Thus, psychoanalysis has led me to re-evaluate a little, not only myself, but also my ambitions, my visions, what I want with things. It has been useful, I think. […] I have had very high demands on myself throughout my life […] I had to be very good at everything. […] Today I do not have quite as rigid demands on myself as before. [Ingvar, termination]

Some kind of urge, but whose urge was it really? […] Before, my life consisted in constantly being challenged. I have grown up with this. […] I had a lot of public roles, so to speak. I lectured a lot and I taught a lot. At parties I sang, it was very much me in the limelight. […] What I have now is what has been left when the other things disappeared. […] I couldn’t endure to live up to that. […] Nowadays, I have no need to attract attention. I take it very cautiously; I don’t want to have any expectations on me. I have no need to please anyone either, and that’s pretty nice. [Ingrid, follow-up]

### 3. The Self

#### 3.1. More Mature and Nuanced Self-Understanding and Realistic View of Life

Generally, the anaclitic patients, and, typically, the introjective patients, described changes that indicated more mature and complex views of themselves and of life. In psychoanalysis, they learned to problematize and nuance their immediate perceptions and to have an inner dialogue. This contributed to a more solid ground to stand on, a more realistic and down-to-earth view of life, making them more ready to meet new strains in life. They felt that they no longer deceived themselves and hid behind a mask, becoming more genuine in their feelings and expressions, but they were also being forced to live more like a “sober alcoholic” and “endure the lack of excitation” [Ingrid, termination]. Gaining a deeper self-understanding helped them to view themselves with greater sympathy and to live in a more conscious, balanced, responsible, and independent way.

#### 3.2. Enhanced Self-Regard and Ability to Attend and Care for Oneself

Typically, patients in both groups expressed how they got more insight in their own self-destructive patterns of behavior and self-punishing or careless habits. In psychoanalysis, they gained a new desire to care for their health and well-being, becoming better at “mothering themselves” and at assuming responsibility for their own welfare.

#### 3.3. Increased Ability to Accept and Value Oneself

Typically, anaclitic patients, and, as a variant, introjective patients, described how they could more easily admit to themselves and reconcile themselves with different aspects of their personality. They could start psychoanalysis with a desire to “become a new person” [Irma, termination] but instead learned to deepen their self-understanding, to accept their negative traits, to be less self-critical, and no longer be haunted by shame and guilt. They described increased self-esteem and a better ability to value themselves as they were, despite flaws and shortcomings. They felt more mature and genuine, less helpless, stronger, and more generous.

#### 3.4. Improved Reflective Capacity, Experiential Processing, and Resilience

Typically, anaclitic patients, and as a variant introjective patients, described an improved capacity to reflect upon themselves. They felt they were more thoughtful, more able to sort out different qualities in their reactions, to harbor emotions, to make inner and outer events less abstract and more comprehensible, and to meet difficulties one at the time, not rushing into action. They experienced themselves as more stable, resilient, ready to reconcile with the past, and to manage the unpredictability of the future.

#### Comparison

Unlike the domains of Love and Work, we found no distinctive patterns for the anaclitic and the introjective patients within the domain of *The Self*. Accordingly, we found no between-group differences in the frequencies of categories (Table 1). Instead, patients in both groups described similar experiences of gaining more mature and nuanced perspectives on themselves and of life, being able to live a more balanced life, taking better care of themselves, and assuming responsibility for their own well-being. Abandoning illusions and hopes of “becoming a new person,” patients generally learned to accept and value themselves as they were, became reconciled with the past and with different aspects of their personality, able to give vent to their experiences and sorrows, to sort out and harbor emotions, and to reflect on themselves. This made them more anchored in themselves, stronger and more stable, resilient and responsible, happier and more able to appreciate the good life, and confident in their ability to manage future challenges.

Today, I try to encounter difficulties one at a time. I feel more at peace with what has been and how it is now, and then I can face new problems or opportunities in another way, I have some sort of faith that I can cope with this at least. I couldn’t do this before. […] Nowadays, I rather try to view things from different perspectives, testing different ways to look at it. To feel “how does it feel?” and then let it rest in me. I feel I can harbor things in a different way; I don’t need to ward off or try to mitigate it at once. I think I’m more balanced, feeling harmonious in a way that makes me capable. [Angelica, termination]

I have gained a greater ability to have a dialog with myself, and to parry situations, so to say. And then, when I can identify or discern why I’m sad and clarify it to myself, I’m able to let it be so. I regard this as the most important thing, to allow and accept things as they are, and then try to do the best out of things, I try to do the best possible for myself in that particular situation. So this is a strong capability. [Irma, follow-up]

## Discussion

The present study aimed to investigate the initially anaclitic and the initially introjective patients’ experienced changes in the areas of love and work. Both the anaclitic and the introjective patients experienced changes in both areas and typically described less issues related to their predominant personality configuration. However, one of the main findings in our study was the symmetrical, but opposite, pattern of changes in the two patient groups. For the anaclitic patients, changes in the domain *Love and Relationship* involved *turning inward*, from an excessive dependency and preoccupation with others toward an increased self-awareness, independence, relational self-confidence, and more stable and distinct self-identity. In contrast, their descriptions of changes in the domain of *Work and Achievements* indicated an expansive turn *outward*, from passivity, resignation, and low self-esteem toward becoming more outgoing, self-confident, and daring in their professional or educational undertakings. For the introjective patients, changes in the domain *Love and Relationships* implied turning *outward*, from an excessive preoccupation with self-boundaries, independence, and relational distance toward an increased acceptance of own affiliative needs and responsiveness to others, being more able to establish and deepen meaningful close relationships. In the domain *Work and Achievements*, on the other hand, their descriptions implied turning *inward*, from unrealistic self-standards, excessive striving for achievements and craving for recognition toward increased self-reflection, inner calm, more realistic self-demands, and a more thoughtful, balanced, and slow living. Looking at the quality of the experienced changes, both patient groups seem to describe a decreased preoccupation with issues related to their predominant personality dimension and an increased responsiveness to needs typical of the neglected dimension, and thus they were able to integrate features pertaining to the complementary polarity.

Comparing the anaclitic and the introjective patients’ experiences of change, we found no differences in frequencies of categories with the domain of *The Self*. However, we found four such differences in the domain of *Love and Relationships* and a further four in the domain of *Work and Achievements*. This is consistent with findings from previous quantitative studies (Werbart and Forsström, 2014; Werbart et al., 2017), showing an improved anaclitic–introjective balance in both patient groups after psychoanalytical treatments. Both groups of patients described changes in the domains of Love and Work in directions complementary to their initial personality orientation. In this process, changes in the area of the Self seemed to function as mediators of changes in the areas of Love and Work. For the anaclitic patients, several of the changes within the domain of *The Self*, such as a more nuanced self-understanding, an improved reflective capacity, and experiential processing, might have contributed to their increased emotional stability, ability to deal with difficulties in relationships, and increased self-confidence and self-determination. For the introjective patients, changes within the domain of *The Self*, such as a more mature and realistic self-understanding, improved cognitive capability, and increased self-acceptance, might have contributed to the increased openness toward own affiliative needs, decreased performance-related anxiety, and more adequate need of control.

Our findings indicated the centrality of the area of the Self in the change process. This is consistent with previous studies, suggesting that changes in self-representations precede changes in representations of significant others, and that these changes can predict therapeutic change in terms of psychosocial functioning (Harpaz-Rotem and Blatt, 2005; Blatt et al., 2008). Accordingly, qualitative studies of patients’ experiences of psychotherapy demonstrated the critical role of the therapists’ ability to foster changes in the area of the Self (Elliott and James, 1989; Klein and Elliott, 2006; Levitt et al., 2016; Werbart et al., 2016). However, this does not imply that changes within this area can be regarded as being isolated from changes in the domains of Love and Work. For example, a study on patients’ experiences of change after dynamic interpersonal therapy found that patients appeared to have become more aware and accepting of their own desires and needs and better at caring for themselves (Leonidaki et al., 2016). This indicated improved reflective capacity and ability to mentalize their own and others’ mind.

No new category of change emerged from the follow-up interviews, even if some aspects of change could be more elaborated. For example, becoming more reconciled with one’s history and experiencing a consolidation of insights and both inner and outer changes were often more pronounced at the follow-up. In both groups, the former patients described that they continued to benefit from the time in psychoanalysis and that their changes continued after termination, even if some problems remained. They could describe how psychoanalysis became a part of their inner world:

[…][S]omehow, it all feels more integrated into me. The analysis exists and lives within me. […] [My analyst] and I talked a lot about getting this therapist function within me, that this was the aim: to be able to reason with myself, to have this inner dialog. […] So, I feel this is a part of me; my analysis is a part of my frame of reference and my personality as well. [Angelica, follow-up]

However, positive developments might imply loss and mourning. At follow-up, three patients expressed ambivalence toward their inner and outer changes, experiencing loss of previous ways of being, beyond the point of no return. Anja described that she had to pay a price for becoming more independent and emotionally balanced: “in this destructiveness there was something that was more alive. […] [I]t is hard not being able to feel as wounded […] I am hardened.” Ingrid felt that her life after psychoanalysis became “flattened and boring,” lacking “drive and excitation,” even if she thought “it was false” and valued “not burning herself at all ends.” Inez felt she was no longer “as perfect, faithful, consistent, and dutiful” as before, experiencing this as “a loss of whole my identity.” However, she appreciated changes and thought that her problem was that “everything was so impotent and had to be so perfect.” For the anaclitic patient, the price for positive changes was loss of emotional excitement, whereas the two introjective patients experienced loss of their high ideals and the struggle for perfectionism. Even if the patients described loss and mourning of previous ways of being, they could problematize the meaning of these feelings and consider the changes worth their price.

Previous research has indicated that patients primarily undergo changes *congruent* with the dominant polarity of their personality configuration (Luyten and Blatt, 2013; Werbart and Forsström, 2014). Anaclitic patients appear to primarily improve the quality of their interpersonal relationships, whereas introjective patients primarily show improvements in cognitive ability and reduced clinical symptoms (Blatt and Ford, 1994). Accordingly, in our study, the anaclitic patients described improvements in the area of intimacy and interpersonal relatedness, whereas the introjective patients described more reasonable striving for achievements and autonomy. Features typical to their respective personality styles emerged with relative clarity in the ways in which they tended to express themselves and what they focused on in their descriptions of change. Anaclitic patients leant to describe their previous difficulties and their changes in more passive, reactive terms, focusing on affective aspects. In contrast, introjective patients used more factual terms and described changes in a reasoning way, focusing on concrete actions and behaviors. For example, reduced need of approval from others meant for anaclitic patients feeling more independent and less in need of being confirmed by others to maintain an inner stability and a sense of who they were as persons. For introjective patients, it was a matter of more realistic expectations of achievements and increased ability to be happy and satisfied with themselves. Whereas anaclitic patients tended to describe a more stable and firm control, introjective patients described reduced rigidity and more flexible control. These different patterns of verbal communication are consistent with a previous study showing that anaclitic patients use verbalizations indicating a desire for emotional empathy and support, whereas introjective patients tend to communicate in a more distanced and emotionally controlled way and use verbalizations that facilitate exploration and reinterpretation (Valdés and Krause, 2015).

In the present study, vital to the patients’ experiences were changes in the opposite, *complementary* dimension, turning in the opposite directions in the areas of Love and Work. Thus, our study confirms Blatt’s (2007) and Blatt and Luyten’s (2009) expectation that the therapeutic process can enable patients to become more open to needs typical for the other polarity. Whereas the congruency hypothesis is supported by both quantitative studies (perspective from the outside) and by patients’ subjective experiences, the complementarity of change is supported by applying perspective from within the patients’ experiences, as explored in qualitative studies. It seems that psychoanalysis helped the patients to no longer be stuck or overwhelmed by issues that are predominantly manifested in one domain, either Love or Work, opening for the dimensions of Love and Work to interact more dynamically.

In each individual case, we encountered unique, idiosyncratic links between changes within the three domains. Nevertheless, our results suggested that changes in the area of the Self were associated with finding more adaptive ways of being with others, more adaptive coping with strains in life, gaining an increased self-confidence, both socially and professionally, and improved capacity to tolerate and deal with affiliative and achievement-related needs, as well as with difficulties in these areas. The domain of the Self, as experienced by the patients in the present study, seems to be an area of integration of the two developmental lines, relatedness and self-definition, in the maturational process, as described by Blatt (2008). Furthermore, narratives of both the anaclitic and the introjective patients indicated that they experienced their changes as a developmental process. According to Blatt’s two-polarities model, therapeutic progress results from reactivation of normal psychological developmental processes and new internalizations. Within the therapeutic relationship, old petrified patterns can be challenged, reviving the dialectical synergic interaction between the two fundamental polarities of relatedness and self-definition (Blatt et al., 2008; Luyten and Blatt, 2013). According to Blatt, this involves alternating sequences of *gratifying involvement* (attachment) and *experienced incompatibilities* (separation, or some disruption to a gratifying relationship), which are characteristic for both normal psychological development and a successful therapeutic process (Behrends and Blatt, 1985; Blatt and Behrends, 1987).

To sum up, both the initially anaclitic and the initially introjective patients experienced to varying degrees a better balance between their affiliative and achievement-related needs. For some of them, this was accompanied by a feeling of loss of their previous exaggerated preoccupation with issues related to their initially predominant personality dimension. Furthermore, love and work, as dimensions of change in psychoanalysis, are supplemented by the dimension of the self-identity. Taken together, these findings might contribute to our deeper understanding of the complexity and dynamics of change, as experienced by patients in psychoanalysis.

### Strengths, Limitations, and Further Directions

The findings from the present study were based on rich interview material, and they were analyzed by applying a discovery-oriented systematic qualitative methodology. The patients’ implicit knowledge of their own change processes might have been overlooked had only quantitative methods been used (Klein and Elliott, 2006). However, the number of participants was limited. Furthermore, the interviews were not especially designed for the aims of the present study. The CHAP interviews at termination allowed only retrospective assessment of pre-treatment personality configurations. However, this interview protocol is aimed to explore differences between the patients’ problems and life situation before and after psychotherapy. To increase the validity of PMAI assessment pre-treatment, both patient and therapist interviews were used.

Data analysis focused on descriptions of experienced changes, which might have led to underscoring of unchanged areas or impairments. The majority of participants were women, the patients suffered from long-term, severe symptoms, and were referred from psychiatric outpatient clinics to publicly financed psychoanalysis. Thus, the sample is not representative of typical patients in psychoanalysis. A further exploration of patterns of change, as experienced by anaclitic and introjective patients, requires a prospective design with larger samples in different treatment modalities and an interview protocol directly suited for the questions at issue. Additionally, combining qualitative data with quantitative measures within the three dimensions of change can contribute to a more nuanced picture of different paths to therapeutic change. Analyses of the anaclitic and the introjective patients’ styles of verbal communication, and how changes in this aspect covariate with clinical improvement, can contribute to improved treatment techniques (cf. Fertuck et al., 2004).

### Clinical Implications

Our study has demonstrated the importance of focusing on relevant differences and distinctive patterns for patients with different personality configurations. Initial assessment in terms of relatedness and self-definition might facilitate opportunities for the clinicians focusing on personality-related directions of change as well as barriers to change. The observation that positive changes might be accompanied by a feeling of losing former orientation in life, even if they considered the changes worth their price, suggests the necessity of therapeutic work on mourning previous ways of being. The mediating role of changes in the area of the self confirms the centrality of clinical work on strengthening the patient’s self-observation and self-reflection capability and the sense of self-identity. This work has to take different directions with anaclitic and introjective patients. In the initial phase of psychoanalytic psychotherapy, the therapists’ ability to adjust their orientation on relatedness or self-definition to be convergent their patients’ predominant personality configuration can enhance treatment outcomes (Werbart et al., 2018). In the long run, the therapists’ task is to challenge their patients’ preoccupation with issues pertaining with their initial personality configuration, thus balancing gratifying involvement and experienced incompatibilities.

## Data Availability Statement

The datasets generated for this study are available on request to the corresponding author.

## Ethics Statement

The research project has been reviewed and approved by the Regional Ethical Review Board at the Karolinska Institutet in Stockholm, Sweden (2012/356-31/5). All participants gave written informed consent in accordance with the Declaration of Helsinki.

## Author Contributions

AW was the project leader and principal investigator in the PPSS as well as in the present study, planned and designed the work, was responsible for the acquisition of all the data included, continuously scrutinized data analysis, the interpretation of results, and early drafting, and the preparation of the version to be submitted. AB contributed primarily with analysis and interpretation of the data for the work, early drafting, and with critical revision in the later stages of the work. SL contributed with critical revisions of data analysis and of the manuscript. They have also given final approval of the version to be published and agreed to be accountable for all aspects of the work.

## Conflict of Interest

The authors declare that the research was conducted in the absence of any commercial or financial relationships that could be construed as a potential conflict of interest.
